# Chronic Inhibition of Aggressive Behavior Induces Behavioral Change in Mice

**DOI:** 10.1155/2022/7630779

**Published:** 2022-12-29

**Authors:** Hiroshi Ueno, Yu Takahashi, Shinji Murakami, Kenta Wani, Tetsuji Miyazaki, Yosuke Matsumoto, Motoi Okamoto, Takeshi Ishihara

**Affiliations:** ^1^Department of Medical Technology, Kawasaki University of Medical Welfare, Okayama 701-0193, Japan; ^2^Department of Psychiatry, Kawasaki Medical School, Kurashiki 701-0192, Japan; ^3^Department of Neuropsychiatry, Graduate School of Medicine, Dentistry and Pharmaceutical Sciences, Okayama University, Okayama 700-8558, Japan; ^4^Department of Medical Technology, Graduate School of Health Sciences, Okayama University, Okayama 700-8558, Japan

## Abstract

Suppression of anger is more common than its expression among Asian individuals. Emotional suppression is considered an unhealthy emotional regulation. Most studies on emotional suppression have concluded that suppression adversely affects social outcomes, with approximately 5% of the world's population suffering from emotional disorders. However, anger suppression has not received academic attention, and details of the effects of chronic anger suppression on the central nervous system remain unclear. In this study, we performed the resident–intruder test to investigate the effect of chronic suppression of aggressive behavior in mice using a behavioral test battery and to clarify whether suppression of this aggressive behavior is stressful for mice. Mice chronically inhibited aggressive behavior and lost weight. Mice with inhibited aggressive behavior showed a reduced percentage of immobility time during the tail suspension test as well as no changes in activity, anxiety-like behavior, muscle strength, or temperature sensitivity. This study provides scientific evidence for the effects of chronic aggressive behavior inhibition on the body and central nervous system.

## 1. Introduction

Emotion-related brain structures in the human brain are regulated by complex circuits consisting of the orbitofrontal cortex, amygdala, anterior cingulate cortex, hippocampus, and several other interconnected regions [1]. Both genes and the environment contribute to the structure and function of this circuit [2].

The American Psychiatric Association created the Diagnostic and Statistical Manual (DSM) of Mental Disorders that focuses on expressive anger and aggression. In Asian culture, suppression of anger is more common than its expression [3]. Compared with Western values, Asian values, such as groupism, harmony, and interdependence, promote the suppression of negative emotions to maintain or strengthen social networks [4, 5]. Emotional suppression is a common form of unhealthy emotion regulation [6]. Most studies on suppression have concluded that it adversely affects social outcomes [7]. Approximately 5% of people worldwide suffer from emotional disorders [8]. However, anger control has received little academic attention.

Among the negative emotions, anger is one of the most intolerable and is most closely associated with the development of illnesses, for example, cardiovascular stress responsiveness [9]. When anger is triggered, the sympathetic nervous system is activated, and the sympathetic adrenal medulla system is stimulated [10]. Consequently, the endocrine system is activated, and levels of cortisol, angiotensin, thyroxine, hyperglycemic factors, and hormones from the posterior pituitary gland in the blood are increased. Unfortunately, despite the prevalence of psychiatric disorders due to chronic anger suppression, details of the effects of chronic anger suppression on the central nervous system remain unclear. In experimental animals, it is important to understand the association between anger suppression and the neurobiological basis of behavioral and affective regulation. Establishing an animal model of anger suppression is essential for the treatment and prevention of the disease. Therefore, we investigated, for the first time, the effect of chronically suppressing the aggressive behavior in mice using the residentintruder (RI) test. This experimental method will be useful for creating a mouse model for chronic anger suppression, and simultaneously, it will lead to the establishment of treatments for emotional disorders.

Mating and aggression are innate social behaviors prevalent in mammalian and non-mammalian species and are essential for individual survival and reproduction [11]. The expression of emotions through behaviors related to aggression and anxiety is essential for the effective communication between individuals in society. In addition, we aimed to clarify whether suppression of this aggressive behavior is stressful for mice. Stressful life events can trigger many neuropsychiatric disorders, such as anxiety, depression, and dementia [12]. This study will not only help establish a mouse model for mental disorders by suppressing anger but also help develop a therapeutic drug for mental and physical disorders due to chronic anger suppression.

## 2. Methods

### 2.1. Ethics Statements

All animal experiments were performed in accordance with the Animal Research: Reporting of In Vivo Experiments (ARRIVE) guidelines (http://www.nc3rs.org.uk/arrive-guidelines) and the United States National Institutes of Health (NIH) Guide for the Care and Use of Laboratory Animals (NIH publication number: 80-23, revised in 1996) and were approved by the Committee for Animal Experiments at Kawasaki Medical School Advanced Research Centre, Kurashiki, Japan. All efforts were made to minimize the number of animals used and their suffering. The use of animals was minimized via an experimental design that permits statistically significant changes to be demonstrated with the smallest number of animals per group and the smallest number of groups, consistent with scientific rigor.

### 2.2. Animals

Four-week-old C57BL/6N and 8-month-old Institute of Cancer Research (ICR (CD-1); retired from breeding) male mice were purchased from CLEA Japan (Tokyo, Japan) and housed in cages (C57BL/6N mice: 5 animals per cage, ICR mice: 1 animal per cage) with food and water provided *ad libitum* under a 12-hour light/dark cycle at 23–26°C. These strains are representative of inbred and non-inbred strains. They are also the most widely used strains in various behavioral tests. Considering that behavioral diversity is partially sex-dependent and that comparing the behavior of male versus female mice was not the purpose of this experiment, only male mice were included.

### 2.3. Inhibited Aggression Procedure

The treatment was performed by applying the social defeat stress procedure [13] and RI test [14]. The RI test was designed and validated to study rodent aggression in aggressive male rodents under standardized laboratory conditions [15, 16]. Male territorial consciousness is a well-defined scientific concept; animals mark their area with feces and urine. As a result of territorial awareness, residents attack strange male rodents who have invaded their home cages [11]. In the experiment using social defeat stress, the mouse to be tested is an intruder mouse; however, in this study, a resident mouse was used for the test. One week prior to testing, aggressor ICR mice were screened. Aggression was evaluated by introducing a novel 4-week-old C57BL/6N mouse to each study mouse at an interval of 3 minutes per day for 3 days and assessed by the latency to attack and number of attacks. Only ICR mice that showed aggression on three consecutive days were used in subsequent experiments. Aggression was confirmed when the behavior of biting the intruder 10 times or more in 3 minutes was observed. Prior to repeated tests, ICR mice were housed alone for 2 weeks.

**Figure 1:**
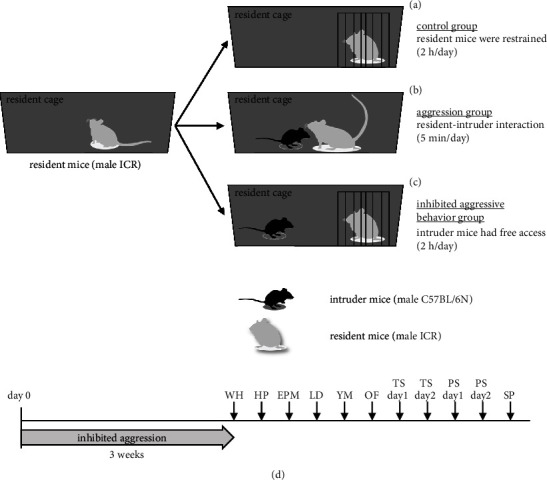
Schematic of the experimental design and time schedules. (a) Control group: a resident ICR mouse is placed into one of the wire cages (7.5 × 7.5 × 10 cm; vertical bars, 0.5 cm apart) that were located in the corners of home cage for 2 hours per day. (b) Aggression group: C57BL/6N mice of the experimental group are placed as intruders in the ICR mouse cages for 5 minutes per day. During the contact process, C57BL/6N mice were attacked by ICR mice, exhibiting evasion, compliance, resistance, and other behaviors. The C57BL/6N mice were rotated daily for 3 weeks to ensure that every ICR mouse in the experimental group had the opportunity to show aggression to different intruders. (c) Inhibited aggressive behavior group: a resident mouse is placed into one of the wire cages that were located in the corners of home cage. The intruder C57BL/6N mice were placed in the ICR mouse cages for 2 hours per day. C57BL/6N mice were allowed to move freely in home cages with wire cages containing resident ICR mice. The C57BL/6N mice were rotated daily for 3 weeks to ensure that every ICR mouse in the experimental group experienced stress from different intruders. (d) Animals in the control, aggression, and inhibited aggressive behavior groups were subjected to tests once a day for 3 weeks. Afterwards, we performed the behavioral test battery. Mice were subjected to one behavioral test per day. ICR, Institute of Cancer Research; WH, wire hang test; HP, hot plate test; LD, light/dark transition test; YM, Y-maze test; TS, tail-suspension test; PS, Porsolt forced swim test; SP, sucrose preference test.

ICR mice were randomly divided (http://www.randomizer.org) into control, aggression, and inhibited aggressive behavior groups (Figures 1(a), 1(b), and 1(c)). Over 3 weeks, the three groups experienced different tests (Figure 1(d)). Three weeks later, the mice were subjected to behavioral tests. (1) In the control group, a resident ICR mouse was placed into one of the wire cages (7.5 × 7.5 × 10 cm; vertical bars, 0.5 cm apart) that were located in the corners of the home cage for 2 hours per day. (2) In the aggression group, C57BL/6N mice in the experimental group were placed as intruders in ICR mouse cages for 5 minutes per day. During the contact process, C57BL/6N mice were attacked by ICR mice, exhibiting evasion, compliance, resistance, and other behaviors. C57BL/6N mice were rotated daily for 3 weeks to ensure that every ICR mouse in the experimental group had the opportunity to show aggression to different intruders. (3) In the inhibited aggressive behavior group, a resident mouse was placed into one of the wire cages located in the corners of the home cage. Intruder C57BL/6N mice were placed in ICR mouse cages for 2 hours per day. C57BL/6N mice were allowed to move freely in their home cages with wire cages containing resident ICR mice. C57BL/6N mice were rotated daily for 3 weeks to ensure that every ICR mouse in the experimental group experienced stress from different intruders. Considering that chronic restraint stress on mice is 2 hours per day, the stress period was set to 2 hours in this study [17].

### 2.4. Behavioral Tests

All behavioral tests were conducted in rooms between 09:00 and 16:00 during the light phase of the light/dark cycle. Each test was separated from the next by at least 1 day. We tested the mice randomly. After testing, the equipment was cleaned with 70% ethanol and super hypochlorous water to prevent artifacts caused by lingering olfactory cues. Tests were performed in naïve mice in accordance with the test order described later. At the end of the study, animals were sacrificed via carbon dioxide inhalation.

### 2.5. Wire Hang Test

During the wire hang test, a mouse was placed on a wire mesh that was then inverted and waved gently so that the mouse gripped the wire. A wire hang test apparatus (O'Hara & Co., Tokyo, Japan) was used. Latency to fall was recorded.

### 2.6. Hot Plate Test

The hot plate test was used to evaluate nociception (sensitivity to a painful stimulus) [18]. Mice were placed on a plate heated to 55.0 ± 0.3°C, and latency to the first paw response (foot shakes or paw licks) was recorded. A latency period of 30 seconds was defined as complete analgesia and was used as the cutoff time to prevent tissue injury.

### 2.7. Elevated Plus Maze Test

Anxiety-like behavior was examined using the elevated plus maze. The apparatus consisted of two open arms (8 × 25 cm) and two closed arms of the same size and material (white plastic plates), with 30-cm-high transparent walls. The arms were elevated to a height of 40 cm above the floor. Arms of the same type were located opposite to each other. Each mouse was placed in the central square of the maze, facing a closed arm, and was allowed to move freely between the four arms for 6 minutes. The mice were video-recorded, and the number of arm entries, distance traveled (m), and time spent in the open arms were analyzed using the ANY-MAZE software (Stoelting Co., Wood Dale, IL, USA).

### 2.8. Light/Dark Transition Test

The light/dark transition test was performed as previously described [19, 20]. The apparatus consisted of an acrylic cage (22 × 44 × 40 cm) divided into two sections of equal sizes by a partition with a door. One chamber had white acrylic walls and was brightly illuminated (200 lx) by lights above the ceiling of the chamber; the other chamber had black acrylic walls and was dark (50 lx). Both chambers contained white plastic floors. Mice were placed in the dark chamber and allowed to move freely between the two chambers for 6 minutes with the door open. The distance traveled (m), total number of transitions, and time spent in the light chamber (seconds) were analyzed using the ANY-MAZE software.

### 2.9. Y-Maze Test

Spatial working memory was measured using a Y-maze apparatus (arm length, 40 cm; arm bottom width, 3 cm; upper arm width, 10 cm; and wall height, 12 cm). Mice were placed in the center of the Y-maze for 6 minutes. Visual cues were placed around the maze and remained constant throughout the testing sessions. The mice were tested with no previous exposure or habituation to the maze. The total distance traveled (m), number of entries, and number of alternations were recorded and analyzed using the ANY-MAZE software.

### 2.10. Open Field Test

Exploratory behavior, anxiety-like behavior, and general locomotor activity were examined using an open field test. Each mouse was placed in the center of an apparatus consisting of a square area surrounded by walls (45 × 45 × 40 cm). The distance traveled (m), number of entries into the central area, and time spent in the central area (seconds) were recorded [21]. The central area was defined as the middle 20 × 20 cm^2^ portion of the field. The test chamber was illuminated at 100 lx. Data were collected over a 30-minute period; analysis was performed using the ANY-MAZE software.

### 2.11. Tail Suspension Test

Depressive-like behavior was examined using the tail suspension test. Each mouse was suspended by its tail 60 cm above the floor in a white plastic chamber using adhesive tape placed <1 cm from the tip of the tail. Their behavior was recorded for 6 minutes. Images were captured using a video camera, and the immobility time was measured. The “immobile period” was defined as the interval when the animals stopped struggling for ≥1 second. Data acquisition and analysis were performed using the ANY-MAZE software.

### 2.12. Porsolt Forced Swim Test

The Porsolt forced swim test was also used to examine depressive-like behavior. The apparatus consisted of four Plexiglas cylinders (height, 20 cm; diameter, 10 cm). The cylinders were filled with water (23°C) to a depth of 7.5 cm [22]. The mice were placed in the cylinders for 6 minutes, and their behavior was recorded. The immobility time was evaluated using the ANY-MAZE software.

### 2.13. Sucrose Preference Test

Before testing, mice were allowed to adapt to two bottles of solution (plain water and 1% sucrose solution) for 2 days [23]. Then, the two bottles were removed for 16 hours overnight. The mice were separated individually per cage and presented with two bottles for 6 hours in their home cages. The two bottles were weighed before and after the test. Sucrose preference was calculated as the ratio of the weight of sucrose solution consumed to the weight of total fluid intake.

### 2.14. Statistical Analyses

Statistical analyses were conducted using the SPSS software (IBM Corp., Tokyo, Japan). Data are presented as box plots. No statistical methods were used to determine the sample size. Data were tested for normal distribution using Bartlett's test. For three-group comparisons, one-way analysis of variance (ANOVA) (for normally distributed data) and Kruskal–Wallis tests (for non-normally distributed data) were used to determine statistical significance. Differences were regarded as statistically significant at *p* < 0.05.

## 3. Results

### 3.1. Effect of Inhibited Aggression on Body Weight Gain

**Figure 2:**
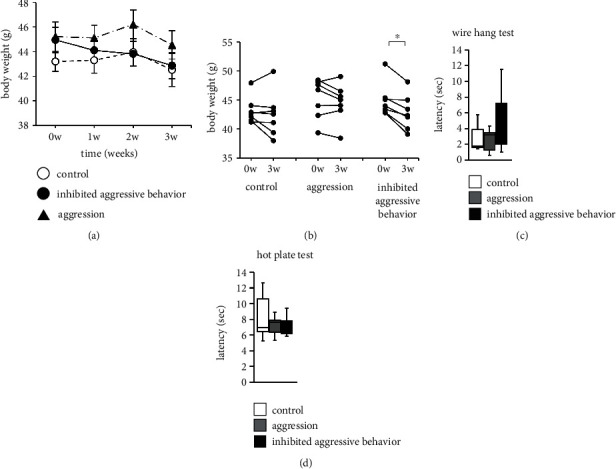
Physical characteristics of the aggressive behavior-inhibited mice. (a) Body weights of mice are weighed weekly for 3 weeks. (b) Individual results of the body weight change for 3 weeks. (c) Latency to fall in the wire hang test. (d) Hot plate test results. Data are presented as mean ± standard error (a) or box plots (c) and (d). ∗*p* < 0.05 and ^+^*p* < 0.1. (a)–(d) control group: *n* = 7, aggression group: *n* = 7, and inhibited aggressive behavior group: *n* = 7.

We compared the body weights of the three groups. The mice were weighed weekly for 3 weeks, and no significant differences were observed in body weight between the three groups (Figure 2(a); Table 1). The change in body weight for 3 weeks was considerably decreased in the inhibited aggressive behavior group (Figure 2(b); Table 1). There were no significant differences in the body weight change for 3 weeks in the control and aggression groups.

### 3.2. Effect of Inhibited Aggression in the Wire Hang and Hot Plate Tests

We compared neuromuscular strength (wire hang test) of the inhibited aggression, aggression, and control groups. There were no significant differences between the three groups (Figure 2(c); Table 1). The mice were placed on a hot plate to assess nociception and evaluate the chronic inhibition of aggressive behavior with thermal pain, and no significant differences in pain thresholds between the three groups were observed (Figure 2(d); Table 1).

### 3.3. Effect of Inhibited Aggression in the Elevated Plus Maze Test

In this study, approximately 90% of the mice fell from the experimental apparatus. Therefore, we could not obtain data from this behavioral test.

### 3.4. Effect of Inhibited Aggression in the Light/Dark Transition Test

**Figure 3:**
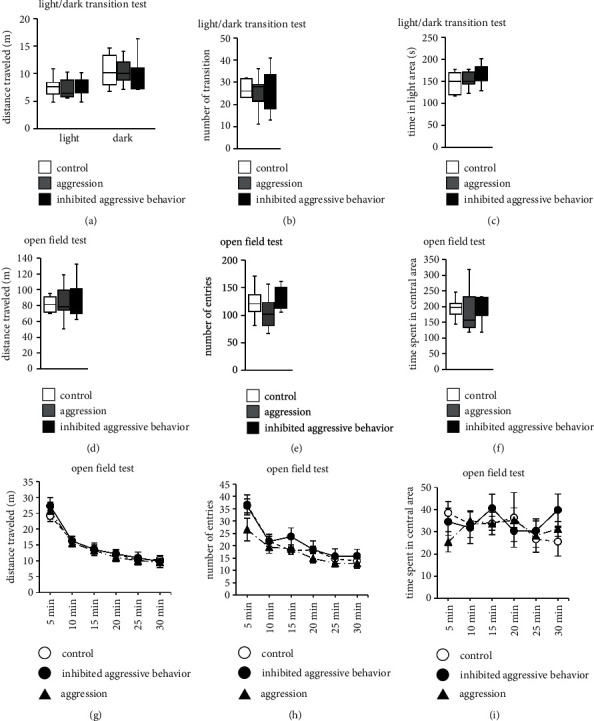
Performance of aggressive behavior-inhibited mice in the light/dark transition test and open field test. Total distance traveled (a), number of light/dark transitions (b), and time spent in the light area (c). Total distance traveled (d), number of entries into the central area (e), and total time spent in the central area (f). Graphs show the distance traveled (g), number of entries into the central area (h), and time spent in the central area (i) in each of the 5-minute test periods. Data are presented as box plots (a)–(f). ∗*p* < 0.05 and ^+^*p* < 0.1. (a)–(i) Control group: *n* = 7, aggression group: *n* = 7, and inhibited aggressive behavior group: *n* = 7.

Anxiety-like behavior was evaluated in the inhibited aggressive behavior group using the light/dark transition test. There were no significant differences in the distance traveled (Figure 3(a); Table 1), number of transitions between light/dark compartments (Figure 3(b); Table 1), or time spent in the light compartment between the three groups (Figure 3(c); Table 1).

### 3.5. Effect of Inhibited Aggression in the Open Field Test

In the open field test, we observed no significant difference in the total distance traveled (Figure 3(d); Table 1), number of entries into the central area (Figure 3(e); Table 1), or time spent in the central area between the three groups (Figure 3(f); Table 1). There were also no significant differences in the distance traveled in each 5-minute period (Figure 3(g); Table 1), number of entries into the central area in each 5-minute period (Figure 3(h); Table 1), and time spent in the central area in each 5-minute period between the three groups (Figure 3(i); Table 1).

### 3.6. Effect of Inhibited Aggression in the Y-Maze Test

**Figure 4:**
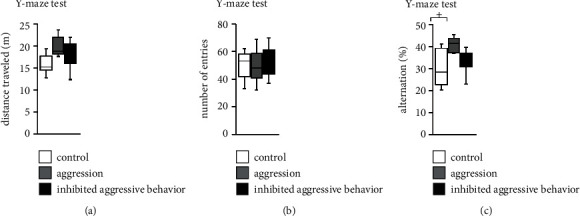
Performance of aggressive behavior-inhibited mice in the Y-maze test. Total distance traveled (a), total number of arm entries (b), and percentage of alternations (c). Data are presented as box plots (a)–(c). ∗*p* < 0.05 and ^+^*p* < 0.1. (a)–(c) Control group: *n* = 7, aggression group: *n* = 7, and inhibited aggressive behavior group: *n* = 7.

We evaluated short-term spatial working memory by monitoring the spontaneous alternation behavior of the inhibited aggressive behavior group using the Y-maze test. We found no significant differences between the three groups in the total distance traveled (Figure 4(a); Table 2), number of arm entries (Figure 4(b); Table 2), and percentage of alternations in the total number of entries (Figure 4(c); Table 2). The aggression group showed a tendency to have an increase in the percentage of alternations in the total number of entries compared with the control group.

### 3.7. Effect of Inhibited Aggression in the Tail Suspension Test

**Figure 5:**
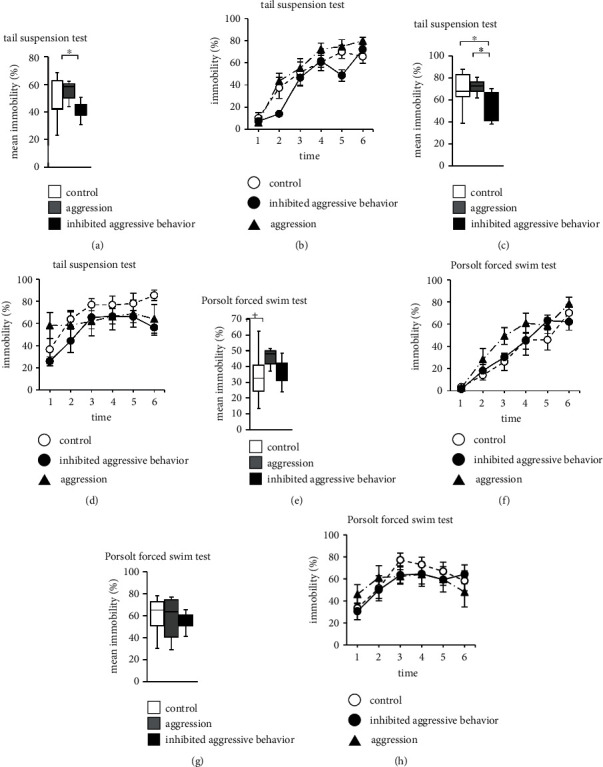
Performance of aggressive behavior-inhibited mice in the tail-suspension test and Porsolt forced swim test. The proportion of total time spent immobile (a), and the proportion of time spent immobile in each 1-minute period (b) on day 1. The proportion of total time spent immobile (c), and the proportion of time spent immobile in each 1-minute period (d) on day 2. The proportion of total time spent immobile (e), and the proportion of time spent immobile in each 1-minute period (f) on day 1. The proportion of total time spent immobile (g), and the proportion of time spent immobile in each 1-minute period (h) on day 2. Data are presented as box plots (a), (c), (e), and (g) or mean ± standard error (b), (d), (f), and (h). ∗*p* < 0.05 and ^+^*p* < 0.1. (a)–(h) Control group: *n* = 7, aggression group: *n* = 7, and inhibited aggressive behavior group: *n* = 7.

We evaluated depressive-like behavior in mice with inhibited aggression using the tail suspension test. The total immobile time for the habituation session on day 1 was considerably lower in the inhibited aggression behavior group than in the aggression group (Figure 5(a); Table 2). There were no significant differences between the three groups with respect to the percentage of time spent immobile in each 1-minute period (Figure 5(b); Table 2). The total immobile time for the test session on day 2 was markedly lower in the inhibited aggression behavior group than in the control and aggression groups (Figure 5(c); Table 2). There were no significant differences between the three groups in the percentage of time spent immobile in each 1-minute period for the test session on day 2 (Figure 5(d); Table 2).

### 3.8. Effect of Inhibited Aggression in the Porsolt Forced Swim Test

We evaluated depressive-like behavior in mice with inhibited aggression using the Porsolt forced swim test. There were no significant differences in the total immobile time (Figures 5(e) and 5(g); Table 2) and percentage of time spent immobile in each 1-minute period (Figures 5(f), 5(h) and Table 2) for the habituation session on day 1 and for the test session on day 2 between the three groups.

### 3.9. Effect of Inhibited Aggression in the Sucrose Preference Test

**Figure 6:**
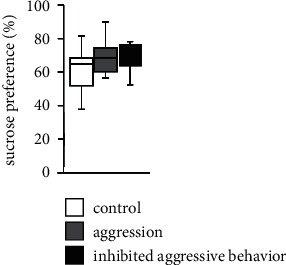
Performance of aggressive behavior-inhibited mice in the sucrose preference test. Percentage of sucrose water consumption at 6 hours in the sucrose preference test. The data are presented as box plots. ∗*p* < 0.05 and ^+^*p* < 0.1. Control group: *n* = 7; aggression group: *n* = 7; and inhibited aggressive behavior group: *n* = 7.

Finally, we evaluated whether inhibition of aggression behavior affects the interest of mice in the sweet solution using the sucrose preference test. The three groups showed a preference for 1% sucrose solution. There were no significant differences in the percentage of sucrose consumption with respect to the total liquid consumption between the three groups (Figure 6**and**Table 2).

## 4. Discussion

In this study, we investigated changes in mouse behavior after 3 weeks of aggressive behavioral inhibition. Mice with inhibited aggressive behavior gradually lost weight compared with mice capable of aggressive behavior. Mice with inhibited aggressive behavior showed a reduced percentage of immobility time in the tail suspension test as well as no changes in activity, anxiety-like behavior, muscle strength, or temperature sensitivity. These results indicate that inhibition of chronic aggressive behavior alters the behavior of mice.

Mice with inhibited aggressive behavior lost more weight than mice in the other groups. Stress changes weight and food intake in animal models, and daily restraint stress caused reduced food intake and weight loss in male ICR mice [17]. Chronic restraint stress has also been reported to reduce weight in other mouse strains [24, 25]. The results of our study suggest that inhibition of aggressive behavior may be stressful for mice. This weight loss may have been due to reduced food intake. Moreover, in this study, the control group mice were placed in a wire cage for 2 hours daily to restrict movement. However, there were no changes in the body weight of mice in the control group, suggesting that the mice in the control group were not stressed.

Wire hanging is ideal for measuring muscle coordination and endurance in mice [26]. No significant differences were found between the three groups in the wire hanging test, suggesting that the inhibition of chronic aggressive behavior does not affect muscle strength in mice.

Analgesia was measured using the hot plate test. This classic assay reflects different modalities of heat nociception [27]. In our experiment, the hot plate test results were not statistically different between the three groups, suggesting that the inhibition of aggressive behavior does not affect pain susceptibility.

The open field test is a behavioral experiment that measures anxiety over a wide area [28, 29], whereas the light–dark transition test is a behavioral experiment that measures anxiety due to a bright place [20, 30]. The elevated plus maze test is a behavioral experiment that measures anxiety due to high places [31–33]. There are several types of anxiety-like behaviors, for example, anxiety due to high places, bright places, and large objects. Mice with inhibited aggressive behavior did not show a decrease in anxiety-like behavior in the open field and light–dark transition tests. In the elevated plus maze test, 90% of the mice in the three groups fell from the experimental apparatus; therefore, measurement was not possible. These results suggest that inhibition of aggressive behavior does not affect anxiety-like behavior in mice.

Moreover, inhibition of aggressive behavior did not affect cognitive function in mice in the Y-maze test. In contrast, mice that were able to behave aggressively tended to have higher cognitive function in the Y-maze test than the control mice. To properly assess how much cognitive function actually improved, it is necessary to conduct other behavioral tests, for example, the T-maze test [34].

Anxiety and depression are thought to have high comorbidity in humans and animals [35, 36]. The tail suspension and Porsolt forced swim tests are widely used for measuring depressive-like behaviors in rodents. In this study, there was a difference in the depressive-like behavior of mice between the results of the tail suspension and Porsolt forced swim tests. It has been suggested that the Porsolt forced swim test result reflects the difference in the swimming ability of each mouse and hypothermia [37]. Therefore, using the tail suspension test, antidepressants and anxiolytics have also been investigated to reduce the immobility time and increase escape behavior [37, 38]. Chronic inhibition of aggressive behavior reduces depressive behavior in mice. In the tail suspension test on the second day, although the mice had a good understanding of the test environment, those with chronically inhibited aggressive behavior showed a decrease in depressive-like behavior. Repeated exposure to social defeat stress has been reported to cause phenotypes, such as strong depression characterized by anhedonia, anxiety, and social avoidance behavior [39, 40]. Chronic restraint stress also increases depressive-like behavior [41]. It is unclear why chronic inhibition of aggressive behavior, unlike other existing stresses, reduces depressive-like behavior. Further research is warranted to estimate what exactly is driving this behavioral phenotype.

Emotion-related brain structures include the cerebral cortex, hypothalamus, and hippocampus. The hippocampus plays an important role in context-sensitive emotional regulation as a regulatory center for higher levels of stress responses [42]. Chronic stress can damage the hippocampus and cause structural and functional changes. The hippocampus is part of the central nervous system, and hippocampal damage has been shown to be associated with specific cognitive impairments caused by diseases of the central nervous system [43]. It is also possible that the chronic inhibition of aggressive behavior, represented as stress, affects the hippocampus of mice, resulting in decreased depressive-like behavior on the first and second days of the tail suspension test.

However, it is possible that there are differences in depressive-like behavior due to differences in mouse strains. In a study comparing non-inbred ICR and inbred C57BL/6 strains of mice, only C57BL/6 mice showed depressive-like behavior with chronic stress loading [44]. Other studies have shown differences between mouse strains in depression-related behavioral models, for example, the sucrose preference [45], forced swim, and tail suspension tests [46]. Here, we used non-inbred ICR mice, which tend to exhibit aggressive behavior [47]. Further studies are needed to determine whether similar results can be achieved by the chronic inhibition of aggressive behavior in C57BL/6 mice. At the very least, this study shows that inhibition of aggressive behavior reduces depressive-like behavior in ICR mice.

Mice that were able to behave aggressively in the forced swim test tended to exhibit more depressive-like behavior than the control mice. No significant difference in the results of the forced swimming test on the second day was confirmed between the groups. The cause of increased depressive-like behavior in the forced swim test is unknown. It is also possible that a 2-hour restraint in the control mice reduced depressive behavior in the forced swim test. Further research is warranted to clarify this point.

In animal studies, the most frequently used approach to assess the ability to experience pleasure is the sucrose preference test [48]. In this study, there was no significant difference in sucrose preference between the three groups. It is possible that the inhibition of aggressive behavior did not affect the sucrose preference of mice, but it is also possible that the effect of the inhibition did not last for 2 weeks after the start of the behavioral test. Abnormal behavior due to social defeat stress is persistent and lasts for at least 4 weeks after the last exposure [49]. Furthermore, the effect of this stressor on behavior is reversible. To analyze sucrose preference in detail, experiments during the treatment period must be conducted.

Aggression encompasses various behavioral patterns and is multidimensional in terms of origin, motives, expressions, and functions [50]. Negative emotions, for example, stress and anger, activate the hypothalamic–pituitary–adrenal and sympathetic adrenal medulla axes and induce the release of pituitary hormones and adrenal hormones, such as the adrenocorticotropic hormone, glucocorticoids, prolactin, growth hormone, and noradrenaline [51]. Aggressive behavior is beneficial for obtaining food, water, and a female spouse (in the case of males). Furthermore, aggressive behavior is beneficial, as it is used to protect one's territory, offspring, or social status. Therefore, the suppression of beneficial behaviors is considered stressful for individuals.

Hwabyeong is a unique phenomenon seen in Koreans that occurs because they have suppressed their anger for a long period. Hwabyeong was listed in the DSM-IV as “culture-dependent syndrome” in 1994 [52]. Hwabyeong develops when anger and unfairness are suppressed and accumulated after exposure to stressful life events [53]. Although aggressive behavior may not represent anger in mice, this study provides a mouse model for Hwabyeong. However, further research is required to investigate its association with Hwabyeong.

In the most common experimental rodents, aggressive behavior is restricted to male rodents. Therefore, one of the serious concerns was the difficulty of using female mice in this study. In the past, the use of female mice has proven difficult in social defeat models. This usually depends on the fact that male mice do not easily display aggressive behavior toward female mice [54]. To investigate the effect of inhibition of aggressive behavior in female mice, it is necessary to establish a new experimental method.

In this study, it is also possible that residents showed other forms of aggression (vocalization/posture) than directly attacking the intruder. To thoroughly establish this as a model, further research is needed to quantify other aspects of behavior during the paradigm.

In this study, chronic inhibition of aggressive behavior showed that mice tend to behave inconsistently, with small abnormalities. Changing the period of inhibition of aggressive behavior or the strain of mice may change the experimental results.

## 5. Conclusions

Chronic inhibition of aggressive behavior reduced depressive-like behavior in mice using the tail suspension test. This study provides a scientific basis for the effects of chronic aggressive behavior inhibition on the body weight and behavior in mice. Additionally, this experimental method provides a new approach to investigate the effect of anger suppression.

## Figures and Tables

**Table 1:** Significance levels for the effects reported in Figures 2 and 3.

						*p*-Value
Figure 2	**A**		control	Versus	aggression	0.674
			control	Versus	inhibited aggressive behavior	1
			aggression	Versus	inhibited aggressive behavior	1
	**B**				control	0.216
					aggression	0.346
					inhibited aggressive behavior	0.004∗
	**C**		control	Versus	aggression	0.417
			control	Versus	inhibited aggressive behavior	0.705
			aggression	Versus	inhibited aggressive behavior	0.24
	**D**		control	Versus	aggression	0.284
			control	Versus	inhibited aggressive behavior	0.193
			aggression	Versus	inhibited aggressive behavior	0.808
Figure 3	**A**	Light	control	Versus	aggression	0.881
			control	Versus	inhibited aggressive behavior	0.747
			aggression	Versus	inhibited aggressive behavior	0.637
		Dark	control	Versus	aggression	0.94
			control	Versus	inhibited aggressive behavior	0.604
			aggression	Versus	inhibited aggressive behavior	0.657
	**B**		control	Versus	aggression	0.422
			control	Versus	inhibited aggressive behavior	0.473
			aggression	Versus	inhibited aggressive behavior	0.931
	**C**		control	Versus	aggression	0.52
			control	Versus	inhibited aggressive behavior	0.124
			aggression	Versus	inhibited aggressive behavior	0.35
	**D**		control	Versus	aggression	0.904
			control	Versus	inhibited aggressive behavior	0.798
			aggression	Versus	inhibited aggressive behavior	0.706
	**E**		control	Versus	aggression	0.252
			control	Versus	inhibited aggressive behavior	0.593
			aggression	Versus	inhibited aggressive behavior	0.101
	**F**		control	Versus	aggression	0.879
			control	Versus	inhibited aggressive behavior	0.7
			aggression	Versus	inhibited aggressive behavior	0.592
	**G**		control	Versus	aggression	1
			control	Versus	inhibited aggressive behavior	1
			aggression	Versus	inhibited aggressive behavior	1
	**H**		control	Versus	aggression	0.756
			control	Versus	inhibited aggressive behavior	1
			aggression	Versus	inhibited aggressive behavior	0.303
	**I**		control	Versus	aggression	1
			control	Versus	inhibited aggressive behavior	1
			aggression	Versus	inhibited aggressive behavior	1

**Table 2:** Significance levels for the effects reported in Figures 4–6.

						*p* value
Figure 4	**A**		control	Versus	aggression	0.251
			control	Versus	inhibited aggressive behavior	0.199
			aggression	Versus	inhibited aggressive behavior	0.885
	**B**		control	Versus	aggression	1
			control	Versus	inhibited aggressive behavior	0.75
			aggression	Versus	inhibited aggressive behavior	0.75
	**C**		control	Versus	aggression	0.084^+^
			control	Versus	inhibited aggressive behavior	0.305
			aggression	Versus	inhibited aggressive behavior	0.451
Figure 5	**A**		control	Versus	aggression	0.305
			control	Versus	inhibited aggressive behavior	0.214
			aggression	Versus	inhibited aggressive behavior	0.031^*^
	**B**		control	Versus	aggression	0.919
			control	Versus	inhibited aggressive behavior	0.641
			aggression	Versus	inhibited aggressive behavior	0.093^+^
	**C**		control	Versus	aggression	0.754
			control	Versus	inhibited aggressive behavior	0.043^*^
			aggression	Versus	inhibited aggressive behavior	0.023^*^
	**D**		control	Versus	aggression	1
			control	Versus	inhibited aggressive behavior	0.63
			aggression	Versus	inhibited aggressive behavior	1
	**E**		control	Versus	aggression	0.099^+^
			control	Versus	inhibited aggressive behavior	0.729
			aggression	Versus	inhibited aggressive behavior	0.183
	**F**		control	Versus	aggression	0.298
			control	Versus	inhibited aggressive behavior	1
			aggression	Versus	inhibited aggressive behavior	0.551
	**G**		control	Versus	aggression	0.721
			control	Versus	inhibited aggressive behavior	0.593
			aggression	Versus	inhibited aggressive behavior	0.858
	**H**		control	Versus	aggression	1
			control	Versus	inhibited aggressive behavior	1
			aggression	Versus	inhibited aggressive behavior	1
Figure 6			control	Versus	aggression	0.194
			control	Versus	inhibited aggressive behavior	0.969
			aggression	Versus	inhibited aggressive behavior	0.207

## Data Availability

Data supporting this research article are available from the corresponding author or first author on reasonable request.
